# Representation of Indigenous peoples in climate change reporting

**DOI:** 10.1007/s10584-017-2076-z

**Published:** 2017-10-03

**Authors:** Ella Belfer, James D. Ford, Michelle Maillet

**Affiliations:** 1 McGill University, Montreal, QC Canada; 2 Priestley International Centre for Climate, University of Leeds, Leeds, UK

## Abstract

This article examines how newspapers reporting on climate change have covered and framed Indigenous peoples. Focusing on eight newspapers in Canada, the USA, Australia, and New Zealand, we examine articles published from 1995 to 2015, and analyze them using content and framing analyses. The impacts of climate change are portrayed as having severe ecological, sociocultural, and health/safety impacts for Indigenous peoples, who are often framed as victims and “harbingers” of climate change. There is a strong focus on stories reporting on the Arctic. The lack of substantive discussion of colonialism or marginalization in the reviewed stories limits media portrayal of the structural roots of vulnerability, rendering climate change as a problem *for*, rather than *of* society. Indigenous and traditional knowledge is widely discussed, but principally as a means of corroborating scientific knowledge, or in accordance with romanticized portrayals of Indigenous peoples. Widespread disparities in the volume, content, and framing of coverage are also observed across the four nations.

**Electronic supplementary material:**

The online version of this article (10.1007/s10584-017-2076-z) contains supplementary material, which is available to authorized users.

## Introduction

Indigenous peoples are widely acknowledged as uniquely sensitive to the impacts of climate change; many Indigenous communities inhabit regions that are already experiencing rapid changes in temperature, weather patterns, and species distributions, with impacts exacerbated by legacies of economic, social, and political marginalization and colonization (Ford 2012; Savo et al. 2016; Wildcat 2013). Efforts to address climate change through adaptation and mitigation are essential, yet there is concern that such actions could perpetuate marginalization and increase vulnerability if they do not reflect the worldviews, needs, and rights of Indigenous peoples (Ford et al. 2016b). Such concern is underpinned by an absence of Indigenous voices in research, policy, and decision-making around climate change at local to global scales (Ford et al. 2016a; Maldonado et al. 2016). Despite this neglect, in recent years, there has been increasing recognition in the global arena of the unique sensitivity of Indigenous peoples, the need to respect Indigenous rights in climate policy, and the importance of Indigenous knowledge systems in responding to climate change, evident in the text of the Paris Agreement and discourse within the United Nations Framework Convention on Climate Change (Ford et al. 2016b).

The growing discursive space around Indigenous peoples and climate change is uneven by region and population, however reflecting different national political circumstances, the nature of the risks posed by climate change, and the extent and nature of engagement by Indigenous Peoples Organizations and communities in climate advocacy (Ford et al. 2016b; Maldonado et al. 2016). Mainstream media also likely influences national and international discourse on Indigenous issues in this context, establishing the salience of climate change as an issue, influencing how the public and policy makers understand and engage with it, and making visible Indigenous experiences of climate change (Boykoff 2012; Carvalho 2010; Schmidt et al. 2013). The role of the media herein is particularly important for Indigenous peoples—who in many cases are geographically isolated and often have less access to institutional power—serving as an important forum from which to broadcast alternative narratives and generate public pressure (Koch-Baumgarten and Voltmer 2010; McCallum et al. 2012). Media coverage of climate change has also been demonstrated to materially impact Indigenous communities, including access to funding streams (Callison 2014; Marino and Lazrus 2015). However, studies reveal pervasive under-representation of Indigenous issues within mainstream media coverage in high-income nations, and the widespread use of frames which perpetuate racist tropes and delegitimize Indigenous actors while masking socioeconomic legacies of colonization (Anderson and Robertson 2011; Rankine et al. 2014).

There is a well-developed scholarship examining portrayals of climate change in the media (e.g., Anderson 2009; Boykoff 2011), yet very little of this work has focused on newspaper coverage of Indigenous peoples. Exceptions include studies of the localized impact of coverage (Huntington 2013; Marino and Lazrus 2015), Roosvall and Tegelberg’s (2013, 2015) analysis of coverage of Indigenous issues at the Copenhagen climate summit (COP15), and Walter’s (2012) examination of how Indigenous peoples are portrayed in environmental coverage in Australia. In light of this dearth of scholarship, this paper identifies and examines the coverage and framing of Indigenous issues in climate change reporting in mainstream newspapers in high-income nations.

## Methodology

### Article selection

The USA, Canada, Australia, and New Zealand were chosen for this case study. These nations provide a particularly salient context to examine and compare coverage of Indigenous issues in climate change reporting, as nations with histories of undermining Indigenous rights at national and international levels (Lightfoot 2010), and as sites with long histories of Indigenous resistance and activism at local to international scales (Lightfoot 2016; Niezen 2003).

Two influential English-language broadsheet newspapers with the largest national circulation from each country were selected; where there were not two national newspapers, the regional paper with the largest readership was chosen (Table S1 ). Using a two-decade timeframe consistent with other long-term studies of climate coverage (Broadbent et al. 2016; Ford and King 2015), articles published between January 1, 1995 and December 31, 2015 were cataloged. The newspapers included were *The Globe and Mail*, *National Post*, *The New York Times*, *Washington Post*, *The Age*, *The Australian*, the *New Zealand Herald*, *The Dominion*, and *The Dominion Post.* All articles (news, op-ed, etc.) were documented using the *Factiva* database. Relevant search terms were then constructed to identify articles with a predominant focus on both climate change and Indigenous peoples (Table S2). Four hundred eighty-five articles were selected for screening.

To screen articles, the title and first paragraph of each article was read to determine relevance, with sentences surrounding the “climate change” term in the article read for additional context as needed. Articles about India, indigenous fauna and flora, or pre-historic climate change were excluded, as were articles where climate change was only one of multiple impacts affecting Indigenous peoples. Where no explicit connection to climate change was made, articles about resource extraction and conservation were excluded, resulting in the removal of several dozen articles. Ultimately, 92 articles were selected for analysis.

### Coding

Within each article, the headline, section, column, and text were coded for content. A combination of frame analysis, drawing on Entman (1993), and conventional content analysis, drawing on Hsieh and Shannon (2005), were used. Content was coded descriptively and for context, in order to provide descriptive explanations (Hsieh and Shannon 2005). Frames were conceptualized as a way to “promote a particular problem definition, causal interpretation, moral evaluation, and/or treatment recommendation” across the entire article (Entman 1993, p. 52). Codes were developed inductively through familiarization and refined through three trial rounds of coding, with five to ten articles coded in each round. A set of codes was developed to describe characteristics for climatic impacts, responses, and agents (See Supplementary Information). The coding scheme captured the stage, scale, and type of impacts and responses, severity of impacts and the type of knowledge used to identify them, and actor types and roles (e.g., victim, researcher). Additionally, thematic codes (e.g., colonialism, resiliency) were developed inductively to identify broader concepts. Finally, a questionnaire provided additional context on each article’s type, the author’s identity, and any major events linked to the article’s publication. Coding was conducted predominantly by one coder using Atlas.ti, with a second researcher coding a sub-sample of texts to ensure replicability and reduce the possibility of bias. Articles coded during trial rounds were re-coded toward the end of the process, and results were compared to verify replicability. Throughout the familiarization and coding processes, a research journal was kept to document potential research biases, trends, and other notes.

### Analysis

The first round of analysis was conducted by examining code frequency, prevalence, and co-occurrence using Atlas.ti, to gain a preliminary understanding of inter-relation of codes and concepts. Descriptive statistics were used to assess which codes were most prevalent and most frequently co-occurring, and to track changes in code prevalence across the sample. These findings informed subsequent analysis, which was conducted using a mixed-methods approach similar to those used in other media analyses (Antilla 2005; Boykoff and Boykoff 2007). Qualitative analysis was conducted by assessing similarities in headlines and article subjects across the sample, and by comparing and contrasting quotations marked by particular codes; the predominant aim of qualitative analysis was to contextualize quantitative findings, draw out broad themes, and assess the prevalence of particular framings within the sample.

## Results

### Portrayal of climate change impacts

The sociocultural, health/safety, and ecological impacts of climate change were discussed most often in the articles reviewed, with almost equal frequency. Fifty-seven percent (80/140) of references to the timing of impacts within articles were coded as “ongoing,” meaning they were described as already having been observed, with the expectation that the trend would continue. The majority of impacts discussed occurred over short timeframes and were identified by Indigenous individuals. Negative and severe consequences of climate change were most commonly reported on, with 81% (105/129) of references to the severity of impacts discussing negative effects, and 69% (72/105) of negative effects described as having a large magnitude; for instance, an article describing the damaging impacts of the pine beetle on the Tl’azt’en people is titled “We might become extinct” (Glavin 2006). Adverse impacts were most frequently described as ongoing, with descriptions such as “ecological mayhem” lending weight to calls to action within these articles (White 2009).

### Scale of focus

A focus on regional impacts was dominant across unique impacts discussed within articles (*n* = 116), with 64% (74/116) of impacts described as occurring at a regional level. Discussion of global- and community-level impacts was less common, with 14 global- and 16 community-level impacts documented. Regional impacts spanned the broadest variety of types, while descriptions of global-level impacts drew links between impacts occurring across regions. Journalists describing community- and individual-level impacts often documented the story or experience of a single Indigenous person as they adapted to―or suffered from―impacts of climate change. For instance, one journalist described a seasoned hunter’s near-death experience of falling through thinning Arctic ice (McIlroy 2007). Articles with a focus on localized impacts personalized the effects of climate change, most often by victimizing profiled individuals.

Where responses to climate change were documented (*n* = 110), the national level was the main level of focus (42/110), with less than a quarter discussing responses at regional (27/110) or global (23/110) scales, and even fewer discussing responses at community (12/110) or individual (5/110) levels. Seventy-five percent (9/12) of community-level responses were adaptation-focused, while 64% (27/42) of responses occurring nationally and 78% (18/23) globally were mitigation-focused.

### Reporting on mitigation and adaptation

Unique mitigation (48%, 53/110) and adaptation (50%, 55/110) responses were profiled in almost equal proportion in the articles reviewed (Fig. S1). Of the adaptation responses discussed, 58% (32/55) were “soft” responses―defined as policy, legal, administrative, institutional, or financial interventions―as opposed to “hard” techno-engineering responses (Fig. S1.1); “soft” adaptation responses included, for example, the imposition of a ban on caribou hunting (White 2010). Notably, many articles examining lifestyle alterations to manage changing conditions explicitly cited the lack of funding for infrastructure development as the primary impetus for pursuing these responses, particularly when discussing relocation of communities. Other lifestyle alterations included the recommendation to revert from snowmobiles to traditional sled dogs for increased safety in light of melting Arctic ice.

Responses to climate change were described as having approximately equal positive and negative impacts. Economic costs were most frequently discussed as negative impacts of both mitigation and adaptation actions. Articles discussing economic costs of responses which predominantly benefited Indigenous communities tended to strongly focus on these costs; for example, an article describing the participation of Indigenous people in an Indigenous environmental health conference was titled “Ottawa pays travel costs for aboriginal delegates” (Curry 2008). Discussion of positive responses varied more widely, with many articles discussing the multiple benefits of a particular response. A similar number of articles had either a positive or negative framing of response options.

### Engagement with Indigenous/traditional knowledge systems

The role of Indigenous or traditional systems of knowledge—collective, place-based bodies of knowledge, practice, and belief accumulated across generations and renewed by each new generation—(Berkes 2008; Cruikshank 2012)—in understanding and responding to climate change is well studied in academic literature (Nakashima et al. 2012). Throughout the articles reviewed, the role of Indigenous or traditional knowledge (IK/TK) in addressing climate change was widely documented. Almost twice as many impacts were identified by IK/TK as by Western or scientific knowledge (70 vs. 42 instances). In approximately 40% (16/41) of the articles where IK/TK identified impacts, impacts were also characterized by scientific knowledge. Impacts identified by IK/TK were predominantly ongoing sociocultural or health/safety impacts, while ecological and economic impacts in the immediate and long-term were generally identified by Western systems of knowledge.

Conflict between IK/TK and scientific knowledge was frequently reported within articles reviewed. Until 2007, this was primarily in the context of Indigenous communities describing ongoing impacts of climate change, while scientists and politicians continued to debate how best to respond to climate change. After 2007, conflict was often documented around health assessments of Arctic polar bear and caribou populations, with Indigenous individuals arguing that they faced substantial economic and social costs due to imposed hunting restrictions based on faulty science. Where conflicts between forms of knowledge occurred, observations by Indigenous individuals that favored action on climate change were generally portrayed favorably, while observations undermining the importance of conservation initiatives were always discussed in the context of the economic interests of Indigenous communities.

While the role of Indigenous peoples in identifying impacts was evident in the articles reviewed, their role in decision-making about appropriate responses was less clear. Approximately one third of responses explicitly addressed the integration of IK/TK, the majority being adaptation-focused. Until 2007, responses involving IK/TK were either initiatives launched by Indigenous communities or actions calling for incorporation of IK/TK into decision-making processes; from 2007, several responses incorporated IK/TK, but predominantly by including Indigenous individuals into existing processes, such as invitations to UNFCCC meetings. However, the vast majority of responses from 2007 to 2015 remained either calls for greater inclusion of Indigenous perspectives or independent initiatives launched by Indigenous communities; for example, one article profiled a phone line launched by Inuit to allow hunters to dispute the USA’s ban on polar bear hunting (Inuit launch hotline 2010).

Finally, though most of the discussion around IK/TK noted its increasing importance, six articles framed such knowledge as having been key to historic resilience and survival, but as being no longer relevant to adapting to ongoing climatic changes, or as not being able to adapt quickly enough. These impacts, described on community or individual scales, were most often identified by Indigenous elders or seasoned hunters who are portrayed as deeply knowledgeable about the land, but are ultimately overwhelmed by the impacts of climate change. One elder, speaking about shifting weather patterns, said “We cannot pass on our traditional knowledge, because it is no longer reliable” (Struck 2006). However, this portrayal of IK/TK provided a small counter-current to the general trend: 30% (28/92) of articles analyzed discussed the increasing validation of IK/TK among academics and politicians. Headlines such as “Indigenous weather know-how sits alongside science” (Leung 2007) reveal the widespread coverage of the importance of IK/TK across the sample.

### Marginalization, colonialism, and responsibility

The majority of references to colonialism were implicit; for instance, one article profiling the “chronic problems in native society” only briefly mentioned a cultural resurgence of practices “[…] banned a century ago by missionaries” (d’Oro 2006), but provides no substantive or explicit discussion of these events and their present-day impacts. Across the sample, 25% of articles (23/92) contain references to colonialism, despite no explicit use of the term across the sample. Of these articles, 39% (9/23) discuss treaties between Indigenous nations and federal governments, 22% (5/23) discuss issues related to Indigenous rights and governance, 17% (4/23) describe the impacts of early colonization, and 17% (4/23) refer to the impacts of colonization as ongoing. For instance, one person quoted describes the impacts of the New Zealand Emissions Trading Scheme (ETS) as “another confiscation” from the Māori (Oliver 2008).

However, the interpretation of these implicit references is complicated by a lack of historical context within coverage. Within New Zealand, for instance, articles covering a political conflict over decreased value of land returned to Māori under treaty negotiations mentioned the relevant treaty, but failed to give any historical context for the transfer of Māori land into the hands of the Crown. Similarly, an article on the Navajo nation noted the legislative “complexities” caused by Navajo sovereignty, and the socioeconomic marginalization of communities covered, in a way that implicitly devolved responsibility for marginalization to the “independent” communities (Barringer 2007).

Discussions of marginalization were far more frequent and substantive than discussions of colonization, with 45% of articles (41/92) referencing marginalization. These articles discussed political marginalization (i.e., exclusion from decision-making processes), economic marginalization (i.e., unemployment, lack of access to institutional funding), and social marginalization (i.e., racism, unsafe living conditions). While some articles explicitly linked marginalization with heightened vulnerability to climatic impacts, marginalization was rarely historically contextualized, with few articles or individuals discussing the need to address such underlying conditions in responding to climate change. Few explicit links between a lack of government support and ongoing marginalization are made.

When discussing responsibility for the severity of climate change impacts, and for impacts of subsequent responses, responsibility was evenly attributed to Indigenous communities and developed nations (18 articles vs. 17). However, the content of assigned responsibility varied significantly. When discussing the culpability of developed nations, articles targeted a generalized notion of industrialized society. The first attribution of responsibility for climate change is found in a series of articles covering an unsuccessful lawsuit launched by Inuit against the USA in the Inter-American Commission on Human Rights, arguing that the USA’s failure to ratify Kyoto was directly linked to the destruction of the Inuit way of life (Watt-Cloutier 2015). In contrast, articles assigning responsibility to Indigenous communities tended to focus on specific individuals, communities, or groups (e.g., hunters); for example, one journalist wrote that “Southern researchers can find themselves denied permission to work in specific northern regions if their findings could result in legal limitations to local resource use,” implicitly suggesting Inuit communities were responsible for endangering polar bear populations by resisting hunting bans (Friis-Baastad 2009). While the responsibility of “industrialized society” is discussed in abstract of concrete solutions, the discussion of Indigenous vulnerability to climate change is always contextualized by discussion of tangible alternatives (e.g., relocation into larger cities, acceptance of quotas), implicitly suggesting Indigenous communities are responsible for the severity of climate change impacts that they experience.

### Variation in coverage across nations

The frequency, tone, and content of coverage varied substantially across the four nations in the sample. Coverage in New Zealand was the most distinctive: newspaper articles rarely discussed the impacts of climate change, focusing predominantly (68%, 19/28 articles) on political debates between the Māori Party and other political parties over climate change legislation. Articles with headlines such as “Māori Party laughing all the way to the bank” and “$50m deal buys Māori vote on emissions” overtly placed blame on the Māori Party (Maori Party laughing 2009; Trevett 2009). Other articles explicitly denied the possibility that Māori were experiencing climate change differently than non-Indigenous New Zealanders (e.g., Venter 2000). The tone of these articles contrasts sharply with two articles by New Zealand newspapers profiling the impacts of climate change on Inuit communities, which discussed at length the sociocultural and ecological impacts of climate change (Connor 2005; Morton 2013).

Six articles were screened from the selected Australian newspapers. Three of these discussed potential impacts of government initiatives on Indigenous communities, but included few Indigenous voices or perspectives (e.g., McKenna 2015). Two articles profiled initiatives to share IK/TK with other Indigenous communities and non-Indigenous Australians (Feneley 2013; Leung 2007), while another profiled Inuit experiences of ice melt in the Arctic (Inuit give cold, hard facts on warming 2007).

American coverage often profiled ongoing issues within communities (50%, 7/14 articles), rather than responding directly to major events. Articles from 2002 to 2006 portray Inuit as “sentries” of climate change in order to frame climate change impacts as real and ongoing (e.g., Struck 2006). The remaining articles either personify ongoing impacts as experienced by members of one community or explore the tensions between resource extraction and the need to act on climate change. Coverage centers predominantly on Inuit and Navajo communities, profiling two very different sets of impacts and response to climate change. Articles predominantly featured individuals and communities responding to impacts, with a smaller focus on interactions between the federal or state governments and these communities.

Canadian newspapers provided the most regular volume of coverage (48%, 44/92 of all articles), largely focusing on the Arctic. This included multiple profiles of activist Sheila Watt-Cloutier, ongoing coverage of the ramifications of the USA’s polar bear hunting ban, and the failed Inuit human rights lawsuit against the USA. As compared to other coverage, Indigenous leaders and individuals were more often framed as the centerpiece of articles. Indigenous issues were also framed as integral to questions of resource management, particularly in the context of debates over resource extraction. The issues profiled in Canadian newspapers did not vary substantially from the early 2000s until 2015, with a predominant focus on documenting Inuit experiences of climate change. For instance, “Arctic natives learn the meaning of sunburn” written in 2000, and “In the land of surfers arrives a cold Inuit message: Climate change is real,” written in 2011, have a similar focus and framing (MacKinnon 2000; York 2011).

Across the countries, there is a strong focus on the Arctic (51%, 47/92 articles), with Inuit specifically mentioned in 43% (40/92) of articles. While 80% (32/40) of articles about Inuit were written by Canadian newspapers, newspapers from the three other countries all covered Inuit experiences of climate change, which were often described as “harbingers” of future impacts (e.g., d’Oro 2006). The second most-covered group were the Māori, mentioned in 18 articles, all but one of which were written by New Zealand newspapers. The bulk of coverage centered on political conflicts between the Māori Party, a minority party with several MPs, and other political parties. Thus, coverage largely focused on political figures, as opposed to individual Māori communities. Few other Indigenous groups received substantial coverage, with the Gwich’in mentioned in five articles, and the Navajo and the Dene in three.

### Temporal trends in reporting

**Fig. 1:**
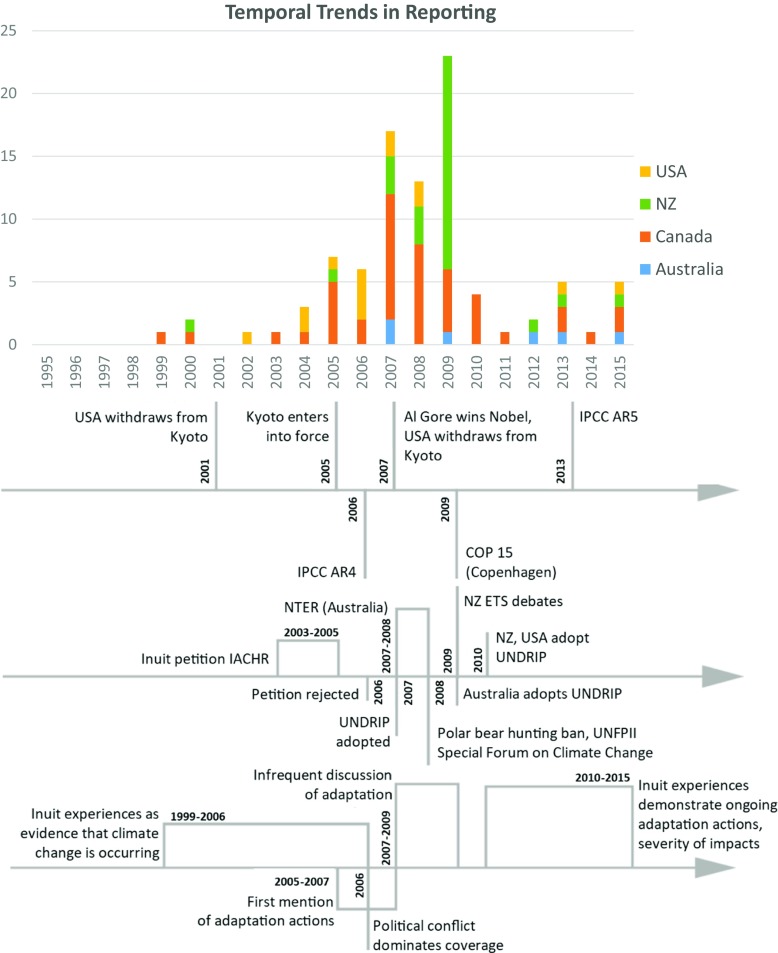
Temporal coverage trends

Three periods of coverage can be discerned between 1995 and 2015, which are broadly consistent across coverage in all four nations (Fig. 1). The first period, 1995–2004, has limited coverage, with only eight articles documented. Of these eight articles, three describe Inuit experiences of climate change (e.g., MacKinnon 2000), three discuss an attempt by the Inuit to sue the US government (e.g., Boyd 2003), and one notes the lack of attendance of Māori at consultations on the implications of the Kyoto Protocol (Venter 2000). Where response options are discussed, these articles predominantly focus on global mitigation action.

In the second phase, 2005–2009, coverage accelerates significantly, with 72% (66/92) of the reviewed articles published in these 5 years. Two trends account for this peak: controversy over the implementation of carbon trading legislation in New Zealand, and a general increase in attention to climate change in national and international spheres. In North America, conflicts over the polar bear hunting ban and the Inuit lawsuit against the US government captured the attention of journalists. Indeed, 50% (33/66) of articles in this period covered political conflicts between Indigenous communities, governments, and environmental groups. Coverage of the ongoing impacts of climate change on communities increased in volume from 2005 to 2009. Discussion of mitigation remained predominant in this period (36 articles), although increasing focus on profiling adaptation actions is also evident (26 articles).

During the third phase, 2010–2015, the volume of coverage declined notably in all four countries. The articles here predominantly profile ongoing impacts of climate change, local adaptation initiatives, and interactions between federal governments and Indigenous communities. The adaptation initiatives profiled included a hotline to dispute the polar bear ban by the USA, and the first World Indigenous Conference (Feneley 2013; White 2010). The prevalence of headlines such as “In the land of surfers arrives a cold Inuit message: Climate change is real” and “Arctic peoples first to feel climate effects” (Morton 2013; York 2011) reveal a continued focus by media in all four countries on mobilizing Inuit experiences to portray the impacts of climate change.

## Discussion and conclusion

As a “public arena,” the media has the potential to shape public understanding of Indigenous issues in a changing climate. While the media can serve as an important forum for Indigenous peoples to challenge dominant narratives (Koch-Baumgarten and Voltmer 2010; McCallum et al. 2012), the few studies examining environmental coverage reveal frequent mis- and under-representation of Indigenous peoples (Roosvall and Tegelberg 2013, 2015; Walter 2012). Impacts of framing and coverage choices may be particularly heightened, because it has been argued that mainstream media plays a primary role in providing information to non-Indigenous peoples about Indigenous issues (Elder 2007; Stolper and Hammond 2010). This study examines the content and framing of Indigenous issues within climate change reporting in mainstream newspapers in high-income nations. We document 92 newspaper articles from the last 20 years focusing on Indigenous peoples in a climate change context across Canada, USA, Australia, and New Zealand, with the number of articles published peaking between 2005 and 2009. Several overarching themes emerge and are examined here.

Firstly, the portrayal of Indigenous peoples as victims of climate change is commonly used to argue for urgency for mitigation action. With coverage of the Inuit human rights case as the epitome, observations and stories provided by numerous Indigenous peoples create compelling personal accounts of the negative consequences of climate change, place climate change as a real and already-occurring phenomenon, and support calls for mitigation measures; these framings echo the role of “intermediaries of urgency” found by Roosvall and Tegelberg (2013, p. 392), as well as long-standing framings of Indigenous peoples as both victims and “noble environmentalists” (Proudfoot and Habibis 2015; Walter 2012). While adaptation actions are profiled across the sample, they are portrayed as isolated incidences and are often used to highlight the difficulty of responding locally to climatic impacts. Thus, Indigenous suffering is used to “sell” the importance of overarching mitigation efforts to the general public, supporting initiatives that do not materially address vulnerabilities of Indigenous communities to climate change. The importance of including Indigenous peoples in mitigation decision-making is ignored by most coverage in the sample.

Secondly, limited reference to colonialism, marginalization, and the history of Indigenous communities in the articles reviewed decontextualizes Indigenous experiences and silences the role of broader sociopolitical factors within which vulnerability to climate change is created and sustained (Cameron 2012; Ford 2012). This omission narrows the types of responses discussed in articles and frames, with articles rarely highlighting the importance of addressing underlying structural root causes of vulnerability. In this way, climate change is constructed as problem *for* society as opposed to a problem *of* society, mirroring broader scientific discourse around Indigenous peoples and climate change (Ford et al. 2016a), and obscuring colonization’s tangible impact on mitigation and adaptation responses (Callison 2014; Marino and Lazrus 2015).

The lack of substantive consideration of legacies of colonization may further result in an implicit devolution of responsibility onto Indigenous communities. For instance, articles which merely note that communities have built permanent settlements in remote, fragile locations, or which discuss the pursuit of additional government funding without historic context, omit critical historical context and therefore promote interpretations that place blame on Indigenous communities. For example, where expensive capital-intensive adaptations are discussed in media articles focusing on the Arctic, the focus is typically on the unrealistic high costs involved but rarely on the re-settlement that in many instances that forced communities into inhabiting such vulnerable locations (Marino and Lazrus 2015). In the USA, the common characterization of Indigenous communities as “nations” without acknowledgement of colonization implicitly characterizes impacts and responses as localized problems, with no substantial linkages to broader US policies or emissions (Barringer 2007; Krauss 2013). In New Zealand, though political controversy explicitly revolves around treaty negotiations, accusations of “special interests” pursued by Māori are bolstered by a failure to address legacies of colonialism (e.g., Trevett 2009). Such insinuations may be reinforced by the widespread use of frames that delegitimize Indigenous actors in broader media coverage across all four nations studied (Drache et al. 2016; Lam et al. 2015; Leavitt et al. 2015; Rankine et al. 2014).

Thirdly, while IK/TK is frequently discussed in the articles reviewed, it is done so within a narrow context. For example, IK/TK was mainly documented where it easily corroborates scientific knowledge, or when the impacts it identifies are sociocultural, and thus beyond the purview of “scientific” research. In such interpretations, complex knowledge systems are reduced to simple observations, valuable because they originate from regions where scientific data is sparse or confirm scientific findings. A focus on Indigenous belief systems, cosmologies, and alternative ways of knowing and interpreting climate change, are largely absent from the articles reviewed, with similar observations made for incorporating IK/TK into Western systems of knowledge (Ford et al. 2016a; Smith and Sharp 2012). Moreover, IK/TK is predominantly valued where it reinforces the romanticized notions of Indigenous peoples present in broader environmental coverage (Walter 2012). Where individuals conflict with such stereotypes, they are implicitly rendered “less” Indigenous, and in cases where conflicts between science and IK/TK are documented scientific knowledge is framed as more impartial and trustworthy (e.g., polar bear controversies in the Arctic).

Fourthly, the differences in volume, timing, content, and tone of coverage across the four nations are striking. Disparities in volume of coverage, and particularly the lack of Australian coverage, could be indicative of differences in the journalistic norms of national newspapers, or of national interest in the subject (See Supplementary Information). However, higher volumes of Canadian coverage may also be consistent with findings that Canada has one of the highest national news shares for climate change coverage as a whole (Stoddart et al. 2015), in addition to the higher relevance of Arctic impacts as the region most dramatically impacted by climate change. Interestingly, the tone of coverage is drastically different in coverage of “foreign” communities. In New Zealand and Australia, coverage of Inuit experiences of climate change is much more sympathetic in tone than in “domestic” coverage, and coverage of Indigenous communities in the Amazon by American newspapers discussed the need for greater incorporation of Indigenous peoples in climate negotiations, a claim that is never made in American “domestic” coverage.

Cross-country comparison also reveals tensions in the understanding of the “citizenship” of Indigenous peoples. With a strong focus on community-level responses by Indigenous nations, the impacts experienced within the continental USA are portrayed as localized issues, rather than the responsibility of the broader American populace or the federal government. Discussions of sovereignty occur most prominently when responsibility for climate impacts is assigned to Indigenous communities, such as the state’s inability to prevent the Navajo nation from building a coal plant (Barringer 2007), or allegations that the Māori Party manipulated treaty rights to strike a political deal (Oliver 2008). In New Zealand, criticism of the Māori Party and of initiatives to include Māori in government decision-making reveals a predominant journalistic discourse portraying Māori as “ordinary” New Zealanders seeking special benefits from government. Indeed, the existence of the Māori Party allows journalists to frame discussions as conflicts between two political parties, while avoiding substantive discussions of marginalization.

While the impact of media coverage on specific communities impacted by climate change has been well-documented (e.g., Callison 2014; Marino and Lazrus 2015), this paper is one of the first to examine the framing and content of reporting on Indigenous issues in national climate change reporting over a broad scale and timeframe. While journalistic norms may have impacted the number of articles returned by the search terms—which focused on the headline, section, and column of the article (See Supplementary Information)— this study is a key in developing baseline insights. Key areas for future research include expanding the scope of the search terms; broadening the focus to examine how Indigenous issues in a changing climate are captured in media from low and middle income nations, in media with diverse political leanings, and in local/regional media; examining in greater depth the link between media representation and the perception of the public and decision-makers on Indigenous issues; and focusing more broadly on coverage of environmental issues in general. As the discursive space around Indigenous peoples and climate change continues to grow, and calls for inclusion of Indigenous peoples and perspectives into climate policy-making increase, it is critical to understand the role that the media plays as a “public arena” in shaping these discussions.

## Electronic supplementary material

ESM 1 (DOCX 70 kb)
