# Supplementary material for: Representation of Indigenous peoples in climate change reporting
Source: Clim Change. 2017 Oct 3;145(1):57–70. doi: 10.1007/s10584-017-2076-z (PMC6560471; doi:10.1007/s10584-017-2076-z)
Supplement: Supplementary file 1 — (DOCX 70 kb) [file 10584_2017_2076_MOESM1_ESM.docx]

**Supplementary Information**

**Figure S1: Response Type over Time**

**Figure S1.1: Adaptation Response Type over Time**

**Figure S1.2: Mitigation Response Type over Time**

**Figure S2: Response Stage**

**Evaluating methodology:**

While the search procedure employed was systematic and extensive, there is also potential that some articles were not captured. In the construction of the ‘Indigenous content’ search term, for instance, the exclusion of more specific names of individual communities, or terms used by indigenous peoples themselves reflected space limitations in the search term, and a judgement that journalists were more likely to use names recognizable to non-Indigenous peoples in their reporting, but may have limited the scope of results. Despite a systematic attempt to modify search terms to reflect multiple spellings of words, a failure to capture some spellings may have also limited the number of results. Most critically, the decision to limit the ‘Indigenous content’ search term to the headline, section and column of the article drastically reduced the number of articles returned, creating a feasible scope for the study, but may have also limited results from newspapers where journalistic norms favor headlines that do not explicitly refer to Indigenous peoples. Thus, a study conducted with a search term that removes this restriction may serve as an important site of future inquiry. The expansion of similar search terms to local and regional newspapers was not feasible within the scope of this study, but could deepen understanding of differences in discourse between and within nations. Finally, the extension of the methods used in this analysis to articles about Indigenous peoples and other environmental issues, such as debates over resource extraction or conservation efforts, may reveal broader patterns of representation in environmental coverage.

**Table S1: Inclusion/Exclusion Criteria**

|  | **Inclusion Criteria** | **Exclusion Criteria** |
| --- | --- | --- |
| **Timeframe** | Jan 1 1995 - December 31 2015  Note: *Dominion* and *Australian* began publishing during 1995. However, the earliest screened article was written in 1999, so it is reasonable to assume that this had no substantial impact on results. | Before January 1995 or during 2016. |
| **Type of newspaper** | Two national English-language broadsheet newspapers, chosen based on circulation in the most recent survey available, and their influence. Where there are not two national newspapers to meet this criteria (Australia), the regional paper with the next largest readership was chosen.  (Newspapers Canada 2014; Roy Morgan Research 2016; Audit Bureau of Circulation 2016; imn, 2014).  Online archives available on Factiva, paid subscription papers.  Note: *The Age* switched from a broadsheet to a compact format in 2014. It was chosen above the *Canberra Times*, the only other Australian broadsheet newspaper, because its readership is approximately ten times larger.  For the U.S., the *Wall Street Journal* was excluded because of its business focus, and the *Washington Post* was chosen over *USA Today* because of its reputation as a higher-quality, publicly-influential newspaper. | Tabloids, online-only, free papers, newspapers from other nations, non-daily newspapers, other language, alternative media, business or financial newspapers, regional newspapers (with the exception noted). |
| **Which newspapers?** | **New Zealand:**  The Dominion (Dec 1995- July 2002), The Dominion Post (July 2002- present),  New Zealand Herald (1994-now)  **Australia:**  The Age (1991 - present)  The Australian (July 1996 - present)  **USA:**  The Washington Post, New York Times  **Canada:**  Globe & Mail, National Post |  |
| **Type of articles** | News, op-ed, editorial, letters to the editor, features | Images, academic articles, advertisements, online/web-only content. |
| **Content screening** | Articles that reference specific indigenous communities | Articles predominantly about indigenous or native flora, fauna, species in the context of climate change |
|  | Articles that focus on the generic impacts of climate change on indigenous peoples without reference to particular communities or impacts | Climate change listed as one of many impacts affecting an aspect of indigenous peoples’ lives. |
|  |  | Repeat stories within same paper (or same corporation) |
|  | Articles about resource extraction, when linked explicitly to climate change. | Articles about indigenous issues where no explicit connection is made to environmental issues or climate change (eg. MMIW) |
|  | Articles about national and international policy, when explicitly linked to the impacts of climate change on indigenous communities. (Eg. an op-ed written by an Inuit person arguing for the adoption of Kyoto to prevent the melting of sea ice in the Arctic). |  |
|  | Articles citing past adaptation of indigenous peoples to climatic change when link to current climate change adaptation is made explicitly. | Articles about the impacts of non-anthropogenic climate change (eg. climate change 100k years ago, end of the last ice age, etc.) |

**Table S2: Search Term**

| “Climate change term” | ("climate change" OR "global warming" OR clim* w/4 chang*) |
| --- | --- |
| “Indigenous term” | AND hl=(“first nation” OR “first nations” OR aboriginal* OR aborigine* OR indigenous OR indian or indians OR native* OR triba* or tribe* OR band council or reservation OR reserve or peoples or algonqu* OR Atha?as?an OR atikamekw OR beaver OR blackfoot OR carrier OR cayuga OR chilcotin OR Chippewa* OR cree OR dakota OR dene OR eskimo* OR gitksan OR gwich’in OR haida OR haisla OR halkomelem OR heiltsuk OR innu OR inu$ OR iroquoi* OR kaska OR kootenay OR kutenai OR ktunaxa OR kwakiutl OR lillooet OR maliseet OR metis OR métis OR michif OR mi’kmaq OR micmac OR Mohawk* OR montagnais OR naskapi OR nisga’a OR Nuu-chah-nulth OR oji-cree OR ojibway OR okanagan OR onondaga OR oneida OR salish OR sarcee OR seneca OR sekani OR shuswap OR siou* OR slavey OR squamish OR stoney OR straits OR tahltan OR thompson OR tlicho OR tlingit OR tsimshian OR tutchone OR wakashan OR wetsuweten or aleut* OR Apache* OR arapaho OR blackfeet OR cherokee* OR cheyenne OR chickasaw* OR Chippewa* OR Choctaw* OR Colville OR Comanche OR Creek* OR Crow OR Delaware* OR Flathead OR Hopi OR Houma OR Gila OR Kiowa OR Lenape OR Lumbee OR Menominee OR Navajo* OR Osage OR Paiute OR Pima OR Potawatomi OR Pueblo OR Seminole OR Shoshone OR “Tohono O'odham” OR Ute OR Yakama OR Yaqui OR Yuman OR Yup’ik OR Pacific-Islander OR Māori or Maori or Ngā* or Waikato or Tūhoe or Te or iwi or Hapū or Hauraki OR Tūranganui OR Waikato OR Rangitāne OR Patukirikiri OR Whakatōhea OR Rongowhakaata OR Rongomaiwahine OR Taranaki OR Tangāhoe OR Pakakohi OR Moriori OR Waitaha OR Koori$ or Ngunnawal OR Goori$ OR Murrdi or Murri or Nyungar or Nunga$ or Nyoongah or Yamatji or Wangai or Nunga or Anangu or Yapa or Yolngu or Bininj or Tiwi or Anindilyakwa or Pal?awa$ OR “Torres Strait Islander” or Ngarrindjeri or Mer)  (2013 Census iwi grouping profiles 2013; 2013 Census iwi individual profiles 2013; Appendix D: Mother tongue and home language 2015; Norris et al. 2012). |

**Table S3: Articles analyzed**

| **Title** | **Author** | **Publication Date** | **Newspaper** | **Content Summary** |
| --- | --- | --- | --- | --- |
| Glacial melting allowed ancient discovery: Natives agree to scientific tests on hunter's remains found on remote B.C. traditional land | Jill Mahoney | 25 August 1999 | The Globe and Mail | The retreat of glaciers reveals ancient human remains, analyzed by scientists with the permission of indigenous leaders. |
| Arctic natives learn the meaning of sunburn: Global warming causes startling shift in weather | Mark MacKinnon | 25 March 2000 | The Globe and Mail | Profile of impacts of climate change on individuals living in Nunavut, need to take mitigation action. |
| MORE OFFICIALS THAN MAORI AT HUIS. | Nick Venter | 20 November 2000 | The Dominion | Poor attendance at meetings held to brief Maori on implications of the Kyoto Protocol. |
| Signs of Thaw in a Desert of Snow; Scientists Begin to Heed Inuit Warnings of Climate Change in Arctic | DeNeen L. Brown | 28 May 2002 | The Washington Post | Inuit testimonials of changes in the climate are being confirmed by scientists and researchers. |
| Climate an issue of rights, Inuit say | Christine Boyd | 11 December 2003 | The Globe and Mail | Inuit launch human-rights case against the United States for failing to ratify the Kyoto Protocol |
| Eskimos Fret as Climate Shifts and Wildlife Changes | Clifford Krauss | 6 September 2004 | The New York Times | Indigenous individuals and scientists describe shifting climate in Nunavut |
| Inuit charge that U.S. threat to their existence | unknown | 15 December 2004 | The Globe and Mail | Inuit launch human-rights case against the United States for failing to ratify the Kyoto Protocol |
| Eskimos Seek to Recast Global Warming as a Rights Issue | Andrew C. Revkin | 15 December 2004 | The New York Times | Inuit launch human-rights case against the United States for failing to ratify the Kyoto Protocol |
| Warming likened to apocalypse: Polar bear, Inuit already doomed, conference told | unknown | 3 February 2005 | National Post | Profile of various predictions of climate change impacts made by researchers at a conference |
| Inuit's bid to sue U.S. over climate delayed: Talks rescheduled: Russians unable to get visas to attend meeting | Steven Edwards | 18 February 2005 | National Post | Complications for Inuit to obtain visas from Russia to meet over human-rights case |
| Hollywood stars, Inuit protest global warming | Catherine Cullen | 23 April 2005 | The Globe and Mail | Two celebrities attend demonstration to raise awareness about impacts of climate change in Nunavut |
| Ice Crusade; Hollywood Celebrities Warm to Inuit Climate-Change Worries | Juliet Eilperin | 26 April 2005 | The Washington Post | Two celebrities attend demonstration to raise awareness about impacts of climate change in Nunavut |
| Deep-freeze treasures; Melting ice patches in the Yukon's high-alpine region are giving up ancient aboriginal throwing darts, ochre paint... | Sigrun Maria Kristinsdottir | 14 May 2005 | The Globe and Mail | Glacial retreat reveals numerous archaeological discoveries |
| Inuit life is not as they've known it | Steve Connor | 20 August 2005 | New Zealand Herald | Profile of environmental and socio-cultural impacts of climate change in the Arctic. |
| In Arctic, a search for the right words; Inuit translators, elders to develop terms to describe climate change issues | Bob Weber | 4 October 2005 | The Globe and Mail | Profile of initiative to develop climate change vocabulary in Inuktikut |
| Inuit See Signs In Arctic Thaw; String of Warm Winters Alarms 'Sentries for the Rest of the World' | Doug Struck | 22 March 2006 | The Washington Post | Profile of environmental and socio-cultural impacts of climate change on individuals in the Arctic. |
| ‘We might become extinct'; It's no secret what the pine beetle is doing to B.C.'s trees — millions of hectares are dead or dying. But that's... | Terry Glavin | 22 April 2006 | The Globe and Mail | Temperature increases enable spread of invasive beetle, causing economic losses and threatening the safety and culture of communities. Loss of indigenous land management techniques cited as part of problem. |
| World Briefing Americas: Inuit Climate Change Petition Rejected | Andrew C. Revkin | 16 December 2006 | The New York Times | Inuit human-rights case against the United States is rejected on basis of lack of evidence |
| Advancing Water Poses Threat to Eskimo Villages | unknown | 27 December 2006 | The New York Times | In Newtok, Yu’pik communities threatened by erosion and flooding caused by climate change, face financial and political barriers to relocation |
| A global crusade to save the Great White North; SHEILA WATT-CLOUTIER: She's travelled widely, warning policians that greenhouse gases are... | Anne McIlroy | 29 December 2006 | The Globe and Mail | Profile of Sheila Watt-Cloutier, Inuit activist calling for action on climate change |
| Erosion of Land and Culture Threatens Many Alaska Natives; Relocation of Villages Has Financial and Other Costs | Rachel d'Oro | 31 December 2006 | The Washington Post | In Newtok, residents navigate political and financial barriers in pursuit of relocation of community away from erosion and flooding, fear cultural loss |
| Listening to the North; Canada's indigenous peoples are in touch with the cadence and rhythms of the land, TERRY FENGE says | Terry Fenge | 6 January 2007 | The Globe and Mail | Author reviews books about the Arctic written by indigenous peoples, arguing for need to understand colonial history to better incorporate indigenous insight and knowledge into policy-making |
| Inuit leader nominated for Nobel; Along with Al Gore, Sheila Watt-Cloutier recognized for efforts on global warming | Doug Mellgren | 2 February 2007 | The Globe and Mail | Sheila Watt-Cloutier, Inuit climate change activist, nominated for Nobel Prize |
| Inuit give cold, hard facts on warming | unknown | 2 March 2007 | The Australian | Several indigenous individuals identify climatic impacts, confirming scientific reports. Importance of further scientific research in the Arctic is emphasized. |
| MAORI TO CHALLENGE FORESTRY CLIMATE POLICIES | Andrew Janes | 22 March 2007 | Dominion Post | Maori claim NZ forestry policy will cause drastic reduction in value of their land, violate treaty rights |
| Maori forest owners take on Government over land-use penalties, carbon credits | Brian Fallow | 22 March 2007 | New Zealand Herald | Maori claim NZ forestry policy will cause drastic reduction in value of their land, violate treaty rights |
| Natives hold key to Ontario power; A proposed east-west energy grid can't proceed without their support | Karen Howlett | 2 April 2007 | The Globe and Mail | Chiefs pursue revenue-sharing agreement with government over construction of electric grid that would import electricity from Manitoba to Ontario |
| Greenpeace sideswipes the Inuit | Robert D. Sopuck | 5 April 2007 | National Post | Editorial arguing that Greenpeace’s use of polar bears as symbol for broader climate change concerns hurts Inuit economically |
| Indigenous weather know-how sits alongside science | Chee Chee Leung | 30 April 2007 | The Age | Indigenous calendar included on government meteorology website, in project celebrating indigenous knowledge |
| Sale of Carbon Credits Helping Land-Rich, but Cash-Poor, Tribes | Jim Robbins | 8 May 2007 | The New York Times | For the Nez Perce, planting forests for carbon credits may provide much-needed income, if carbon markets can be stabilized |
| Fur flies in Canada over proposed U.S. hides ban; Measure aimed at preserving species would block hunters from bringing back trophies, but... | Martin Mittelstaedt | 23 June 2007 | The Globe and Mail | U.S. proposes banning polar bear hunting, citing impact of climate change. The ban would severely impact Inuit economies dependent on hunters for income. |
| Using dogs to adapt to global warming; Quebec Inuit should park their snowmobiles and return to dogsledding to negotiate increasingly... | Tu Thanh Ha | 24 July 2007 | The Globe and Mail | Researcher proposes that return to dogsledding may be safer for Inuit due to thinning ice. Several other indigenous responses to thinning ice are profiled. |
| Navajos and Environmentalists Split on Power Plant | Felicity Barringer | 27 July 2007 | The New York Times | Navajo nation plans to build new coal plant to generate wealth in community, stirring opposition from Navajo individuals, environmentalists, and the New Mexico government |
| Inuit environmentalist a top contender for Nobel; Canadian nominated with Gore could jointly share prestigious award for creating awareness... | Martin Mittelstaedt | 12 October 2007 | The Globe and Mail | Sheila Watt-Cloutier, Inuit climate change activist, nominated for Nobel Prize |
| MAORI BUSINESSES URGED TO EMBRACE NEW TECHNOLOGY | Paul Easton | 19 October 2007 | Dominion Post | Maori business associations identify new business opportunities, citing impacts of climate change on traditional Maori industries |
| Indigenous people describe real perils of global warming | Geoffrey York | 14 December 2007 | The Globe and Mail | Profile of side event at Bali climate change conference, where indigenous leaders from across the world describe impacts of climate change |
| I still wonder how he didn't perish in that water'; Inuit hunter Simon Nattaq knows only too well what climate change is doing to the Far... | Anne McIlroy | 22 December 2007 | The Globe and Mail | Profile of Inuit hunter who fell through thinning ice, describes impact on him and his family |
| Fighting for the right to be cold; For the Inuit, the battle against global warming is about human rights – their culture and way of life... | Anne McIlroy | 28 December 2007 | The Globe and Mail | Profile of Sheila Watt-Cloutier, Inuit climate change activist |
| FROM THE ARCTIC TO THE SAHARA: AN INUIT ADVENTURE IN TIMBUKTU | Stephanie Nolen | 12 January 2008 | The Globe and Mail | Inuit and Tuareg artists meet, discuss the impacts of climate change on their communities |
| Native towns at risk of going up in flames; More than 100 remote B.C. communities in danger after pine beetles create huge swath of dry... | Bill Curry | 6 February 2008 | The Globe and Mail | High fire risk for indigenous communities due to pine beetle infestation, request for pre-emptive large-scale tree removal rejected by government |
| Ottawa pays travel costs for aboriginal delegates | Bill Curry | 13 March 2008 | The Globe and Mail | Indigenous peoples from Canada, US and Mexico meet to discuss climate change and build unified strategies to address environmental health concerns. |
| MAORI FILM FESTIVAL TO GO ON THE ROAD | unknown | 27 March 2008 | Dominion Post | Maori film festival features films about the impacts of climate change on Maori, as well as other environmental concerns |
| As Uranium Firms Eye N.M., Navajos Are Wary; As Ore's Prices Rebound, Navajos Are Wary of Return of Industry With Poor Safety Record in Area | Kari Lydersen | 28 March 2008 | The Washington Post | Global warming increases interest in uranium mining, creating conflict between potential economic benefits for Navajo and potentially drastic health/safety impacts |
| Amazon's 'Forest Peoples' Seek a Role in Striking Global Climate Agreements | Alexei Barrionuevo | 6 April 2008 | The New York Times | Indigenous groups from across Latin America build consensus on plan for wealthier nations to compensate conservation of tropical forests, seek greater political role |
| Hello, Dolly; The Gwich'in people of Canada's North have depended for centuries on a colourful fish called the Dolly Varden – which is now... | Elie Dolgin | 12 April 2008 | The Globe and Mail | Indigenous elder helps scientist locate endangered fish, scientist conducts extensive research to aid in species conservation |
| U.S. hunters targeting polar bears while they can; Looming import ban threatens to kill lucrative, but controversial, tradition of chasing... | Katherine O'Neill | 26 April 2008 | The Globe and Mail | U.S. considers banning polar bear hunting, citing impact of climate change, despite conflicting Inuit knowledge about polar bear health. Article details positive economic and health/safety benefits for indigenous communities of polar bear hunting. |
| U. S. moves to protect polar bear; Condemned By Inuit; Declared a 'threatened' species | Joseph Brean | 15 May 2008 | National Post | US declares polar bear “endangered” species, despite Inuit traditional knowledge, Nunavut government contests decision |
| Polar bear politics hurt Inuit | Mary Simon | 17 May 2008 | The Globe and Mail | Letter to the editor: Author argues that newspaper’s characterization of traditional indigenous knowledge is condescending, characterizes US decision as attempt to appease environmental lobby |
| Govt review to answer Maori fears | Paula Oliver | 3 September 2008 | New Zealand Herald | Ngai Tahu launches claim with Waitangi Tribunal that government emission trading scheme lessens value of treaty settlement, government was not negotiating in good faith during treaty negotiations |
| Ottawa's stand at talks hurting native rights, chiefs say; First nations blast Indian Affairs Minister | Bill Curry, Martin Mittelstaedt | 12 December 2008 | The Globe and Mail | Canada’s opposition to including mention of indigenous rights in UNFCCC negotiations draws criticism from First Nations |
| Indigenous input urged | unknown | 20 December 2008 | New Zealand Herald | National science organization says indigenous communities can benefit financially from managing their lands so as to create carbon credits |
| ‘What are we going to do about the bears?'; For the first time, governments, environmentalists, researchers and Inuit are meeting to discuss... | Patrick White | 14 January 2009 | The Globe and Mail | At first national polar-bear roundtable, Inuit and scientists clash over appropriate government policy on polar bear conservation |
| No common ground at first polar bear summit; Inuit leaders defend right to hunt giant mammals and accuse environmental groups of using... | Patrick White | 17 January 2009 | The Globe and Mail | At first national polar-bear roundtable, Inuit and scientists clash over appropriate government policy on polar bear conservation |
| Where there's a will, there's … another will; How evangelical teachings, television and southern movies shape the Inuit ‘way of knowing' is... | Erling Friis-Baastad | 1 August 2009 | The Globe and Mail | Review of book written by journalist about challenges of climate change research in Arctic, including clashes between indigenous peoples and scientists over research. |
| Maori try to pull criticism of emissions trading plan | Claire Trevett | 28 August 2009 | New Zealand Herald | Maori Party tries to withdraw report critical of National Party’s emissions trading scheme, drawing accusations of a political deal being made |
| MAORI PARTY BACKS OFF CRITICISM OF CARBON DEAL | Vernon Small | 28 August 2009 | Dominion Post | Maori Party tries to withdraw report critical of National Party’s emissions trading scheme, drawing accusations of a political deal being made |
| Maori Party links ETS support to benefit boost | Patrick Gower | 16 September 2009 | New Zealand Herald | Maori Party negotiates with National Party for additional benefit payments in order to secure support for ETS |
| Maori Party ETS deal put under microscope | Audrey Young | 18 September 2009 | New Zealand Herald | Benefits such as subsidies for home insulation scrutinized by opposition parties, as Maori Party and National Party negotiate over weakened ETS |
| Maori Party laughing all the way to the bank | unknown | 19 September 2009 | New Zealand Herald | Journalist accuses Maori Party of prioritizing Maori business interests, extracting money from National Party in exchange for supporting weakened ETS |
| D OES THE Maori Party speak the same language as the Nats? After last week's... | Tapu Misa | 21 September 2009 | New Zealand Herald | Article notes miscommunications over precise benefits obtained in negotiations over ETS legislation, questions integrity of Maori Party in negotiating with National |
| Iwi prepare to exploit assets | Julie Taylor | 24 September 2009 | New Zealand Herald | Iwi prepare for impacts of climate change on industry |
| In a changing North, the torch recalls tradition; Threatened by climate change, the Gwitchin of Old Crow reflect on the links between the... | Gary Mason | 5 November 2009 | The Globe and Mail | Olympic flame arrives in remote Gwitchin community facing socio-cultural impacts of climate change |
| EMISSIONS DEAL FOR IWI ON OFFER Government needs Maori Party votes Carbon dating | Colin Espiner, Tracy Watkins | 17 November 2009 | Dominion Post | Article details gains won by Maori during negotiations over ETS with National, National rejects claims of ‘preferential deal’ for Maori |
| Maori Party deal saves climate change bill | unknown | 17 November 2009 | New Zealand Herald | Article details gains won by Maori during negotiations over ETS with National, accusation of ‘preferential treatment’ from other parties |
| EMISSIONS TRADING ACCORD WITH IWI WILL COST TAXPAYERS | Colin Espiner | 18 November 2009 | Dominion Post | Article details potential cost of ETS settlement with the Maori Party for taxpayers |
| National `buying' Maori votes for emissions plan | Claire Trevett | 18 November 2009 | New Zealand Herald | Labor Party accuses National of giving Maori preferential treatment to secure passage of ETS, criticizes lack of transparency |
| Indigenous leaders want ETS bill shelved | Dennis Shanahan | 21 November 2009 | The Australian | Indigenous leaders call for Australian ETS legislation to be deferred until legal position of indigenous lands and rights to trade carbon credits are clarified. Leaders cite exclusion from policy-making process |
| $50m deal buys Maori vote on emissions | Claire Trevett | 23 November 2009 | New Zealand Herald | Final deal with Maori supposedly gives five iwi rights to plant trees on conservation land and  take profits from the carbon credits, cited as worth $50m a year for Maori, in exchange for political support for ETS |
| IWI COUNTING CASH BEFORE DEAL IS FINAL | Colin Espiner | 23 November 2009 | Dominion Post | Article argues that ETS deal struck because Maori threatened legal action, claims of ‘sweetheart deal’ for Maori at cost of taxpayers and environment |
| Goff: Iwi tree deal little help to Maori | Claire Trevett | 24 November 2009 | New Zealand Herald | Article argues that Maori Party ETS deal primarily benefits Maori elite, results in weakened ETS without addressing impacts on low-income households |
| SELLOUT' CLAIMS OVER MAORI ETS DEAL | Tracy Watkins | 24 November 2009 | Dominion Post | Article argues that Maori Party ETS deal primarily benefits Maori elite, results in weakened ETS |
| FREE TRIP OFFERED TO IWI LEADERS | unknown | 24 November 2009 | Dominion Post | Government flies iwi leaders and elders to Copenhagen climate talks as part of official NZ delegation, one of the conditions of negotiations over ETS legislation |
| SWEETHEART DEAL WITH IWI ELITE LEAVES URBAN MAORI JILTED AGAIN | Karl Du Fresne | 24 November 2009 | Dominion Post | Article argues that Maori Party ETS deal primarily benefits Maori elite at cost of marginalized Maori, accuses Maori ‘aristocracy’ of brokering deal that results in ineffective ETS |
| Communities need funds to adapt to Arctic climate change: Inuit leader | unknown | 12 December 2009 | National Post | Inuit leader discusses need for money for communal deep freezers to store game, as global warming reduces the hunting season |
| Inuit launch hotline for polar bear hunters to dispute proposed ban | unknown | 8 January 2010 | National Post | Inuit launch phone line for hunters to call and provide evidence disputing decline of polar bear population |
| A caribou herd in decline – a way of life in jeopardy; Natives' fight for right to hunt mysteriously disappearing species threatens to.. | Patrick White | 5 May 2010 | The Globe and Mail | The Yellowknives Dene First Nation and the Government of the NWT fight over a caribou hunting ban |
| The Inuit of Greenland have weather on their side | Doug Saunders | 2 October 2010 | The Globe and Mail | Comment: Author argues that global warming will benefit Inuit communities in Greenland because of increased access to oil drilling, mining |
| Kunuk's done what no one else has: listened to the elders; The Fast Runner director skips the ‘experts,' going directly to Inuit elders, and... | Guy Dixon | 20 October 2010 | The Globe and Mail | Nunavut filmmaker makes documentary detailing Inuit observations of climate change. Article also details one particular observation disputed by scientists. |
| In the land of surfers arrives a cold Inuit message: Climate change is real | Geoffrey York | 5 December 2011 | The Globe and Mail | Two Inuit hunters attend Durban climate conference, hoping to prompt action despite stalled negotiation processes |
| Capturing carbon, unlocking wealth for Aborigines - EXCLUSIVE - | Mark Schliebs, Patricia Karvelas | 13 July 2012 | The Australian | Profile of an indigenous-owned carbon farming initiatives with the potential to create jobs for indigenous communities |
| Iwi lose out in carbon crash | Brian Fallow | 18 October 2012 | New Zealand Herald | Crash in carbon prices due to cheap imported units disproportionately impacts Maori, opposition parties criticize weakness of ETS |
| Arctic peoples first to feel climate effects | Jaime Morton | 23 April 2013 | New Zealand Herald | Geologist from NZ makes documentary interviewing climate scientists and the Sami people about climate change, aims to increase understanding of impacts |
| Leona Aglukkaq's personal Arctic stake; The Health Minister plans to use her direct knowledge to give Canada's Inuit a real say in... | Paul Koring | 11 May 2013 | The Globe and Mail | Health Minister Leona Aglukkaq discusses her role within the federal government, argues that resource extraction should expand to the Arctic, argues for greater collaboration with indigenous communities |
| Indigenous connect | Rick Feneley | 27 May 2013 | The Age | Coverage of World Indigenous Network Conference, where indigenous delegates from across the globe connect to discuss environmental opportunities and challenges |
| Future of an Industry and a Tribe Hinges on a Coal Export Battle | Clifford Krauss | 15 June 2013 | The New York Times | The Crow Nation is attempting to address impoverishment by exporting their enormous coal reserves. However, this requires construction of new export terminals, which are fiercely opposed by environmental groups and other indigenous communities. |
| Global warming blessing and curse for road to tuk; Forging a future | Jeff Lewis | 10 September 2013 | National Post | Road-building project between Inuvik and Tuktoyaktuk could be transformative as climate change eases access to natural resources, but permafrost melt may hinder construction |
| THE ROAD AHEAD; Jeffrey Jones explores the $300-million highway from Inuvik to Tuktoyaktuk and discovers the project's potential to reshape... | Jeffrey Jones | 18 January 2014 | The Globe and Mail | Road-building project between Inuvik and Tuktoyaktuk could be transformative as climate change eases access to natural resources, but permafrost melt may hinder construction |
| Iwi plan million-hectare forest planting | Isaac Davidson, Claire Trevett | 10 February 2015 | New Zealand Herald | Iwi leaders propose planting a million hectares of forest in exchange for championing of Maori interests in any international climate change deal, greater emission reduction targets |
| On Parched Navajo Reservation, 'Water Lady' Brings Liquid Gold | Fernanda Santos | 14 July 2015 | The New York Times | Responding to drought exacerbated by climate change, one Navajo woman supplies water to those facing extreme water scarcity in her community |
| Climate change tops the agenda of indigenous visits | Michael McKenna | 22 August 2015 | The Australian | Torres Strait Island leaders confront the federal government about the disastrous impacts of climate change on the low-lying islands and government inaction |
| First Nations chief lauds powers in climate plan | Bob Weber | 28 November 2015 | The Globe and Mail | Greater provisions for consultation of and collaboration with First Nations included in new Alberta climate-change policy |
| Climate change threatens traditional Inuit food supply | Dan Joling | 30 December 2015 | The Globe and Mail | Release of report by Inuit Circumpolar Council calls for policies to protect traditional Inuit food and the Arctic environment, cites health and cultural impacts of environmental degradation |

**Table S4: Prevalence of Actor Types**

| Prevalence of Actor Types | | |
| --- | --- | --- |
| Type | Indigenous | Non-Indigenous |
| General population | 12 | 8 |
| Politician / government official | 67 | 80 |
| Researcher / academic | 3 | 28 |
| Activist | 19 | 16 |
| Industry | 10 | 14 |
| Artist | 4 | 1 |
| Elder | 20 | N/A |
| Hunter | 15 | N/A |
| Children / youth | 3 | N/A |
| Other | 7 | 8 (4 celebrities) |

**Table S5: Prevalence of roles**

| Prevalence of Roles | | | **New Zealand** | | **Australia** | | **Canada** | | **USA** | |
| --- | --- | --- | --- | --- | --- | --- | --- | --- | --- | --- |
| Roles | Indigenous | Non-indigenous | Ind | Non | Ind | Non | Ind | Non | Ind | Non |
| General population | 12 | 8 | 0 | 1 | 1 | 0 | 7 | 5 | 4 | 2 |
| Politician / government official | 67 | 80 | 26 | 41 | 3 | 3 | 27 | 28 | 11 | 8 |
| Researcher / academic | 3 | 28 | 0 | 3 | 1 | 1 | 1 | 18 | 1 | 6 |
| Activist | 19 | 16 | 0 | 0 | 2 | 0 | 11 | 10 | 6 | 6 |
| Industry | 10 | 14 | 5 | 4 | 0 | 0 | 5 | 3 | 0 | 7 |
| Artist | 4 | 1 | 0 | 0 | 0 | 0 | 4 | 0 | 0 | 1 |
| Elder | 20 | N/A | 1 | N/A | 0 | N/A | 9 | N/A | 10 | N/A |
| Hunter | 15 | N/A | 1 | N/A | 2 | N/A | 6 | N/A | 6 | N/A |
| Children / youth | 3 | N/A | 0 | N/A | 0 | N/A | 2 | N/A | 1 | N/A |
| Other | 7 | 8 (4 celebrities) | 1 | 0 | 1 | 0 | 2 | 4 | 2 | 4 |

**Sources Cited**

2013 Census iwi individual profiles (2013) Statistics New Zealand. http://www.stats.govt.nz/Census/2013-census/profile-and-summary-reports/iwi-profiles-individual.aspx?request_value=24540#24540. Accessed May 05 2016

2013 Census iwi grouping profiles (2013) Statistics New Zealand. http://www.stats.govt.nz/Census/2013-census/profile-and-summary-reports/iwi-profiles-grouping.aspx. Accessed May 05 2016

4imn.com (2014) 2014 Newspaper Rankings. http://www.4imn.com/topNorth-America/. Accessed May 08 2016

Appendix D: Mother tongue and home language: classifications from 2011, 2006 and 2001 (2015) Statistics Canada. https://www12.statcan.gc.ca/census-recensement/2011/ref/dict/app-ann004-eng.cfm. Accessed May 05 2016

Audit Bureau of Circulation (2016) Press audit results. Auckland, NZ. http://newspaper.abc.org.nz/audit.html. Accessed May 08 2016

Newspapers Canada (2014) Circulation Report: Daily Newspapers 2014. Toronto, Canada.

http://www.newspaperscanada.ca/sites/default/files/2014_Circulation_Report-Daily_Newspapers_in_Canada_FINAL_20150603_0.pdf. Accessed May 08 2016

Norris T, Vines PL, Hoeffel EM (2012) The American Indian and Alaska Native Population: 2010. United States Census Bureau, Washington D.C.

Roy Morgan Research (2016) Australian Newspaper Readership (12 months to September 2016_. Melbourne, Australia. http://www.roymorgan.com/industries/media/readership/newspaper-readership. Accessed May 06 2016
